# SMART Mental Health Project: process evaluation to understand the barriers and facilitators for implementation of multifaceted intervention in rural India

**DOI:** 10.1186/s13033-021-00438-2

**Published:** 2021-02-08

**Authors:** Abha Tewari, Sudha Kallakuri, Siddhardha Devarapalli, David Peiris, Anushka Patel, Pallab K. Maulik

**Affiliations:** 1 George Institute for Global Health, New Delhi, India; 2 George Institute for Global Health, Sydney, Australia; 3 University of New South Wales, Sydney, Australia

**Keywords:** Process evaluation, Mental health services, India, Mixed methods, Community-based services, mHealth, Stigma, Common mental disorders

## Abstract

**Background:**

Globally, mental health problems are a growing public health concern. Resources and services for mental disorders are disproportionately low compared to disease burden. In order to bridge treatment gaps, The Systematic Medical Appraisal, Referral and Treatment (SMART) Mental Health Project was implemented across 12 villages in West Godavari district of the southern Indian state of Andhra Pradesh. This paper reports findings from a process evaluation of feasibility and acceptability of the intervention that focused on a mental health services delivery model to screen, diagnose and manage common mental disorders (CMDs).

**Methods:**

A mixed methods evaluation was undertaken using quantitative service usage analytics, and qualitative data from in-depth interviews and focus group discussions were conducted with stakeholders including primary care physicians, community health workers, field staff and community members. Barriers to and facilitators of intervention implementation were identified. Andersen’s Behavioral Model for Health Services Use was the conceptual framework used to guide the process evaluation and interpretation of data.

**Results:**

In all, 41 Accredited Social Health Activists (ASHAs) and 6 primary health centre (PHC) doctors were trained in mental health symptoms and its management. ASHAs followed up 98.7% of screen positive cases, and 81.2% of these were clinically diagnosed and treated by the PHC doctors. The key facilitators of implementation were adequate training and supervision of field staff, ASHAs and doctors, use of electronic decision support, incorporation of a door-to-door campaign and use of culturally tailored dramas/videos to raise awareness about CMDs, and organising health camps at the village level facilitating delivery of intervention activities. Barriers to implementation included travel distance to receive care, limited knowledge about mental health, high level of stigma related to mental health issues, and poor mobile network signals and connectivity in the villages. Lack of familiarity with and access to mobile phones, especially among women, to accessing health related messages as part of the intervention.

**Conclusions:**

The evaluation not only provides a context to the interventions delivered, but also allowed an understanding of possible factors that need to be addressed to make the programme scalable and of benefit to policy makers.

## Background

Mental health problems are a growing public health concern. Recent studies demonstrated that the burden of disease associated with mental illness is among the highest for all disorders globally [1, 2]. Resources and services for these mental disorders are disproportionately low as compared to disease burden. The World Health Organization (WHO) estimates that 75–85% of people with diagnosed mental health problems in low and middle-income countries (LMICs) do not receive any treatment due to lack of awareness, scarcity of mental health professionals and high levels of stigma related to help-seeking [3–5]. In India, the treatment gap is exacerbated in rural areas due to low literacy levels, poor knowledge about mental health, absent or inadequate mental health services and even fewer trained mental health professionals. Poverty and lack of public transport also reduces accessibility to primary health centres [6]. In order to bridge the treatment gaps for mental disorders, there is a need for innovative healthcare delivery models, but these need to be implementable, acceptable and feasible within the existing public health system. Studies have examined the effectiveness of task shifting [7] and use of electronic decision support system (EDSS) on mental health services delivery [8, 9] and have also been implemented earlier at a smaller scale by our group in 30 villages in a tribal area of India [10, 11]. India’s primary health system in rural settings essentially comprises of a lay village health worker (Accredited Social Health Activists – ASHAs) who is a local community person and has been educated to about tenth grade. She is provided basic training in identifying common ailments and maternal and childcare needs in the villages and refer such cases to the primary health centre staffed by one medical doctor and paramedical staff. Each ASHA caters to about 1000 individuals and is paid a nominal amount each performance-based activity. Primary Health Centres (PHC) cater to 20,000–30,000 population and is managed by a medical doctor, nurse and paramedical staff and administrative officers. The PHCs manage most common ailments of villagers that fall within the catchment area of a PHC and refer the more complicated cases including conditions like mental disorders, to the district hospitals. The Systematic Medical Appraisal, Referral and Treatment (SMART) Mental Health Project was implemented across 12 villages in West Godavari district of the southern Indian state of Andhra Pradesh. This was a before–after study conducted between 2014 and 2019. The intervention comprised a community anti-stigma campaign, with training of lay village health workers and primary care doctors to identify and manage individuals with stress, depression and suicide risk using an electronic clinical decision support system. The technology-enabled mental health services delivery model was specifically designed for the project. This was used for screening, diagnosing, and managing common mental disorders (CMDs) including stress, depression, and increased suicide risk. This paper reports findings from a process evaluation of SMART Mental Health Project exploring feasibility and acceptability of this complex strategy focused on service delivery; screening, diagnosis and management of CMDs.

## Methods

**Fig. 1:**
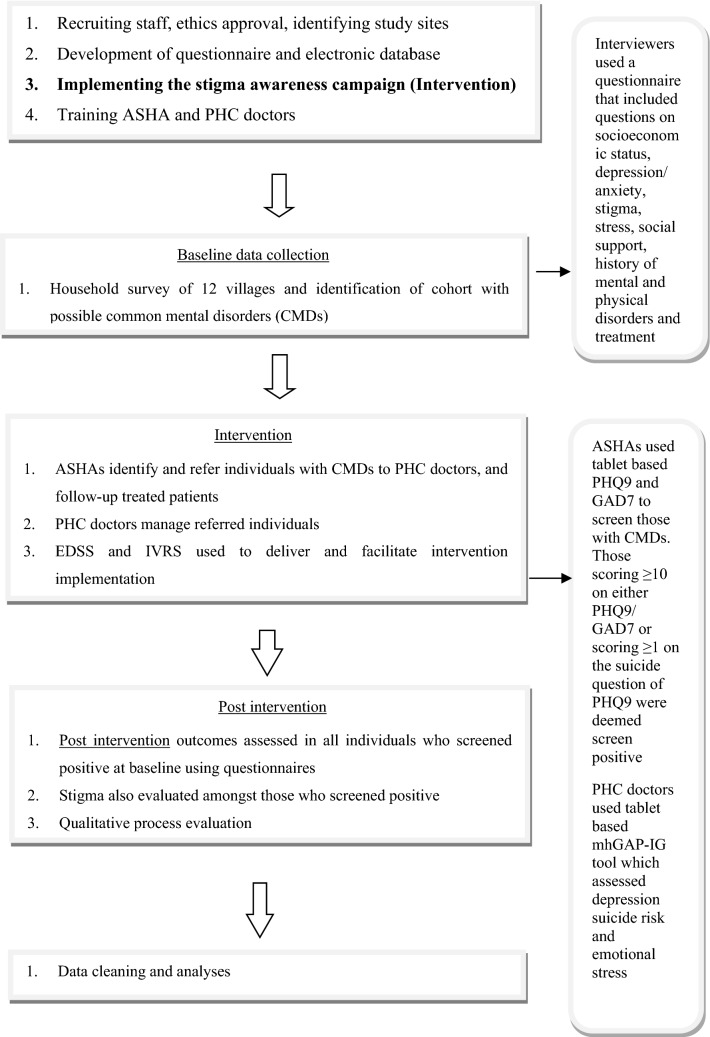
Andersen’s modified behavioural model of health services use. Environment Population characteristics Health Behaviour Outcomes

The project was divided into different stages as outlined in Fig. 1. Altogether 22,046 adults were screened by health workers for increased stress, depression, or suicide risk and 900 were screened positive and referred to a primary care doctor for clinical diagnosis and management. At the end of the one-year intervention phase 843 of them were followed up. The project encompassed a multifaceted intervention consisting of three key components: (1) training of primary care health workers and doctors in screening and managing common mental disorders (CMDs) (which included depression, increased suicide risk and emotional stress); (2) using technology enabled EDSS to facilitate mental health services delivery by primary healthcare workers; and (3) conducting a community-based anti-stigma campaign related to mental health [12].

Table 1 describes the intervention strategies in more detail.

**Table 1:** Specific interventional strategies for SMART Mental Health Project

Key objectives	Development of a multifaceted programme that includes an anti-stigma campaign and the systematic medical appraisal, referral and treatment (SMART) mental health intervention Evaluation of the intervention to provide evidence of feasibility, acceptability and effectiveness of the programme	
Strategies	Training/Educating health professionals (primary healthcare physicians, healthcare professionals (ASHAs) and project staff) with updates in screening, diagnosis and treatment of population for depression, suicidal risk and emotional problems using EDSS tool Educating the community members about mental health and adherence to treatment Offering free screening /counselling services to the community in interventional areas	
Interventional activities based on target groups	General population	Educating about mental health, non-pharmacological treatments of mental illness and the benefits of treatment Sharing brochures and posters on mental health using door-to-door campaign Video shows of a person talking about his own mental illness and a film actor talking about CMD; staging live performances of a drama on mental disorders
	Primary health care Physicians and health care professionals (ASHAs)	Organizing training workshops on the updates in screening, diagnosis and treatment of population for depression. suicidal risk and emotional problems using EDSS tool Training/educational manuals for physicians, and healthcare professionals Knowledge on key symptoms, such as psychotic features, and comorbid conditions, such as drug and alcohol use
	Screen positives and family members	Educating about mental health, non-pharmacological treatments of mental illness and the benefits of treatment Educating patients relatives’ for screening mental illness and raising their sensitization in favor of the patients’ treatment using door to door campaign Organizing health camps for providing free counselling services Referral to the next level of care to access the trained mental health professionals

**Fig. 2:**
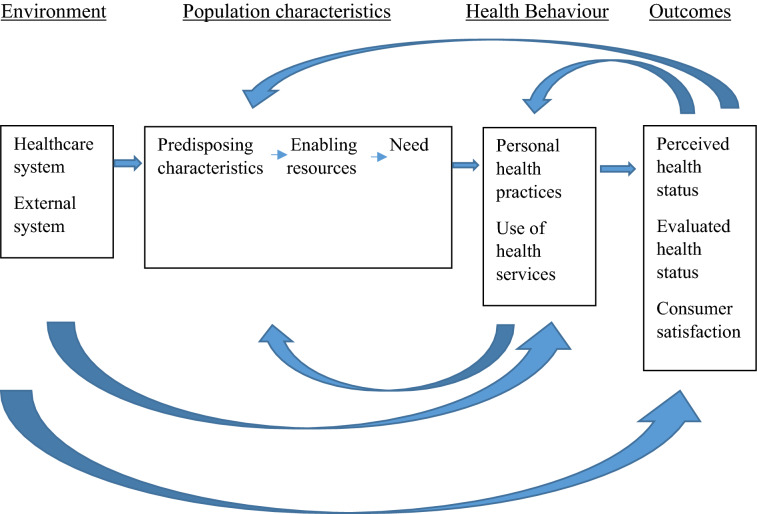
Overall project plan

We adopted the Andersen Behavioral Model for Health Services Use as the conceptual framework to guide the process evaluation [11, 13] (Fig. 2). The key components of the model are environmental, population characteristics, and health behaviour affect outcomes. The outcomes themselves provide a feedback loop that affects perceived need and health behaviour.

### Setting and participants

The data were collected in three primary health centres (PHCs) which serviced the 12 villages included in the study. A diverse sample of resident adults aged ≥ 18 years were recruited ensuring representation from all villages and PHCs. We included both male and female participants and tried to have equal numbers wherever feasible [14]. While details of the method including tools used and cut-off scores have been outlined earlier [12], briefly, the study population were first interviewed using a detailed survey by trained interviewers. This also included questions on depression and anxiety as shown in Fig. 1. However, the same population were rescreened just for depression and anxiety using the same tools, by ASHAs, and those who were screened positive as outlined in Fig. 1 by ASHAs were referred to the primary care doctors. Suicide risk was initially determined based on the depression tool but was also assessed clinically by the PHC doctors. Stress was based on clinical assessment as outlined in Fig. 1.

### Data collection and analysis procedure

*Quantitative data* Service usage on the frequency and type of services used was extracted from back-end data obtained directly from the server. Descriptive quantitative analyses on service usage included activities undertaken, frequency of health care services utilization, type and appropriateness of care provided by doctors, frequency of follow-up of screen positive cases by ASHAs, numbers and success rate of health-related messages sent to screen-positive individuals, and proportion of primary health workers using an Interactive Voice Response System (IVRS) based model. The ASHAs were responsible for monitoring follow-up of screen positive individuals with the doctor and ensure treatment adherence using an algorithm-based traffic light system that allowed them to prioritize follow-up. For example, if the individual had not visited a doctor, questions inquired about the reason; if referral to a psychiatrist was suggested by the doctor, then the ASHAs checked about that in their follow up; if pharmacotherapy or counselling was provided then the questions focused on treatment adherence, social support and stressors. The data not only provided an objective measure of the follow up frequency by ASHAs, but also provided additional information on different factors that enable or discourage people to seek or continue treatment due to their daily life commitments/priorities which made them choose between visiting the doctor vs. earning their livelihood. This information was also captured as a part of regular monitoring of ASHA work by the field staff which was shared periodically by the field staff during the review meetings. The algorithm was based on a 7-in. tablet device. The doctors used an application based on the mhGAP-IG tool which allowed them to diagnose and manage CMDs [15]. This was also set up on a 7-in. tablet in both Telugu and English languages Information about treatment provided could be shared between the doctor and the ASHA tablets. Qualitative data describes the (1) the perceptions of the community members, Accredited Social Health Activists (ASHAs), primary care physicians and other key stakeholders about the intervention and its activities and (2) identifies barriers and facilitators in implementation of the intervention. Qualitative data were collected through in-depth interviews (IDIs) and focus group discussions (FGDs) in local language (Telugu), which were undertaken from December 2016 to March 2017. These were conducted either at village community centres, or at the house of the participants. FGD and IDI guidelines were developed a priori based on the study objectives and Andersen’s theoretical model (Additional file 1: Table S1). Two study team members, a moderator and a note taker, conducted all IDIs and FGDs. The interviews were audio recorded. The data collected through IDI/FGDs were first transcribed verbatim in the local language (Telugu) and then translated into English. Study team members (SK, SD) cross checked all English translated transcripts with transcripts in the local language (also going back to the audio files whenever necessary) to ensure the quality of translation.

FGDs and IDIs data were analyzed inductively to identify and analyse patterns and themes until we reached thematic saturation [16]. The themes were then matched against Andersen’s theoretical framework [13] and the results were outlined using that framework. Data were independently coded by two team members (AT and SK) who followed the phases of analysis outlined by Braun and Clark [17]. The authors (AT, SK, SD, PM) had discussions to identify common themes and to discuss areas of agreement and divergence. NVIVO 9 software (QSR international) was used to code and organise the data [18]. The COREQ guidelines [19] were followed for reporting qualitative research (Additional file 2: Table S2).

**Table 2:** Demographic details of the participant involved in qualitative interviews and Focus group discussions

S. no.	IDI (In depth interviews)	Number of participants (N = 25)	Gender	Age (in years)
1	PHC doctors	n = 5	3 males 2 females	25–64 yrs
2	Community members (Screen positive visited the doctor and were given medical and/or psychological treatment	n = 3	0 male 3 females	24–50 yrs
3	Community members (Screen positive who visited the doctor and were referred to a specialist)	n = 3	1 male 2 females	41–70 yrs
4	Community leaders	n = 13	7 male 6 females	23–74 yrs
5	Key Government official	n = 1	1 female	55 yrs

S. no.	FGDs (Focus group discussions)	Number of FGDs (N = 16)	Gender	Age (in years)
1	ASHAs	n = 2	19 females	25–65 yrs
2	Field Staff	n = 2	9 males 7 females	22–42 yrs
3	Community members(Screen positive by ASHA who visited the doctor)	n = 6	25 males 36 females	25–90 yrs
4	Community members(Screen Positive by ASHA who did not visit the doctor)	n = 3	6 males 13 females	25–80 yrs
5	Community members(Screen Negatives at all phases)	n = 3	11 males 15 females	28–61 yrs

## Results

Quantitative data from backend analytics of different applications was collated and is reported under each theme as supporting information. Qualitative data was obtained from 16 FGDs and 25 IDIs conducted with different stakeholders (Table 2). Twelve FGDs (each lasting for about 1-h) were conducted with 140 community members, separately for males and females. Community members represented a mix 141 of literate (n = 33) and illiterate (n = 53). Majority of adult males were working (n = 32) and most were engaged in agriculture (n = 22). Half of the women were illiterate (n = 32); some were homemakers (n = 26) or were engaged in farming, domestic work (n = 19); and few earned daily 144 wages (n = 8) to supplement their family income. Two FGDs were conducted with ASHAs and one FGD with the field staff. IDIs (each lasting for about 40 min) were conducted with the five PHC doctors and 13 community leaders. One IDI was conducted with a key government official to understand about her perceptions of the intervention (Table 3). The results were analysed against the key themes of Anderson’s Model [13] are outlined below. Each theme consisted of multiple sub-themes relating to specific facilitators and barriers for health service use (Additional file 3: Table S3).

**Table 3:** Identifying barriers and facilitators of mental health services use based on Anderson’s Modified Behavioural Model of health services use

Key components from Andersen’s model	Prior SMART Mental Health Intervention Scenario	Intervention and processes implemented	Participants’ perception about the intervention (barriers, facilitators and recommendations from participants to improve the programme)
Environmental			
Healthcare system	Mental health care was not available at PHC ASHAs lacked knowledge on CMD and skills for interview people on mental health issues Primary care doctors did not have proper knowledge and skills to identify and manage CMD Patients required to travel to distantly located PHCs, leading to financial loss	41 ASHAs and 6 doctors were trained on using the mobile technology-based application 12 Field staff were trained to communicate with ASHAs and doctors and implement the programme and supervise the process ASHAs screened 22,046 community members for CMDs using algorithm based EDSS. Among these, 900 community members were screened positive and referred to PHC for treatment Doctors used this training to manage CMD using mhGAP-IG based EDSS About 104 Health camps organized in villages to facilitate the easier access to doctors and treatment IVRS calls were sent for a period of 1 year to 41 ASHAs, 6 PHC doctors and the screen positives in the community at different intervals and different frequency on a case-to-case basis, to remind screen positive individuals about treatment and to ASHAs about their pending screenings and follow ups In all, 14,849 calls were placed during intervention period; 13,400 call placed to community members;1449 calls placed to ASHAs and doctors;8046 calls to patients	Community members, ASHAs, primary care doctors appreciated the programme and perceived it as valuable in enhancing the knowledge of community members on mental health ASHAs reported that training enhanced their confidence level and interview skills Doctors appreciated the usage of EDSS, as it provided some prior information of the patient and consume less time Almost all ASHAs, and doctors mentioned that poor signal and network connectivity was a barrier on using the applications Training sessions helped ASHAs, doctors and field staff to operate the tablets Distance, lack of public transport facility and financial burden were demotivating factors to access the care Health camps within the locality were appreciated as they reduced time and money spent in visiting the PHC ASHAs paid repeated visits to enquire about health and to motivate them to visit the doctor for treatment Few participants were aware of the IVRS sent to them on their mobile phones Family members owned only one phone set, kept at home Phone sets were owned by their family members, husbands or their children’s; and most of them were illiterate Some people simply assumed that the messages were from service provider and did not even show interest to check these messages at all
Population characteristics			
Predisposing characteristics	Limited awareness about mental health among community members Social issues like poverty, unemployment, and caste system exist in villages	Organized awareness campaign on mental health using several strategies Brochures and pamphlets were used in the door-to-door campaign and community meetings to raise mental health awareness and discuss issues related to stigma; this was repeated 3–4 times in each village A promotional video where a local film actor speaks about mental health and stigma was screened A video of a person with mental disorder to talk about his/her experience was screened and discussed during the campaign Staging a drama by a local theatre group on domestic violence, mental disorder and the need for getting treated	Door-to-door campaign was a key strategy to approach people for face- to face interaction and motivate them for treatment, if required The drama was received very well and there was good participation at all the villages Domestic violence, family restrictions, preoccupation with work were the other reasons which prevented people from visiting the doctors ASHAs were scared to enter in some houses belonged to economically well-off people or to the higher caste Community leaders shared that in some families, the elders did not allow daughters/daughters-in-law to go out for seeking treatment, specifically for mental illness Alcohol addiction and loneliness were perceived as reasons for mental illness Stigma is associated with mental illness, and were highly prevalent
Enabling resources	No pre-existing mental health services in villages Community members were not oriented towards identifying CMD	Local administration and village leaders were informed about the project at each phase ASHAs and doctors used to provide care and treatment Field staff were trained using standard operational procedures and their activities monitored regularly Field staff monitored ASHAs regularly and ensured the quality of data collected by them; supervisors followed up with doctors regularly to check for any problems that they might be facing with the application Health camps in villages enabled patients with CMD to seek care from doctors closer to home	Village leaders reported that people suffering from mental illness needed some support, which they received through this programme Peer learning and sharing of experiences encouraged people to seek health care
Need	Perceived need to seek care for CMDs was negligible as there was no awareness about CMDs ASHAs and doctors were not trained to identify or manage the CMD	Screening of the whole population by ASHAs led to increase in help seeking	Participants suggested more training programs for doctors and ASHAs Most participants recommended that communities need to be more educated about the facts pertaining to mental illness
Personal health practices	Stigma related to mental health was highly prevalent Poor knowledge about CMDs amongst community members and health workers	A campaign was organized to increase mental health awareness and reduce stigma	All participants reported the campaign was beneficial Project increased the awareness about CMDs and the need to seek care
Outcomes			
Use of health services	There was no treatment for CMDs in PHCs	The intervention had a focus on increasing mental health services use for CMDs Task shifting was used to enable mental health care for the rural population Technology driven platforms were used to facilitate provision of mental health services A system developed to ensure follow up by ASHAs and doctors Out of 900 patients, 731 visited the doctor at least once. The doctors were able to deliver the healthcare effectively 104 Health camps organized in villages to facilitate the easier access to doctors and treatment	Programme should be implemented through PHCs, and in collaboration with 104s (ambulance services in the rural areas), assisting them to have access to the nearest treatment facilities
Perceived health status		A comprehensive mental health intervention implemented	The intervention was well received and appreciated in the community
Consumer satisfaction		A pre-post evaluation of the project provided objective assessment of the outcomes	Community members were satisfied with SMART mental health intervention as it led to increase in the knowledge of CMDs in the community

### Environment: healthcare system, external system

Some strategies were introduced to support the healthcare system to enhance mental health services delivery. Namely, training of primary health workers, using of technology enabled mental health service delivery modules.

#### Training of primary health workers

In all, 41 ASHAs and 6 doctors were trained about symptoms associated with CMDs and its management, and the EDSS tool to deliver the intervention. The ASHAs were trained for 10 days (the sessions lasted approximately 7–8 h per day), using a training manual and the doctors were provided an intensive 3–4 h training for 2 days on using the mhGAP tool. In addition, 13 field staff were also given 10 days extensive training on monitoring of the intervention activities. The majority of ASHAs and doctors reported that the training sessions helped them to operate the tablets and record the observations on a regular basis. ASHAs elaborated that training increased their confidence in approaching people to share their mental health problems.

*“It was easy for us to deal with patients as everything was put on the tab, time was saved and work was fast.....otherwise have to write everything on the paper.” (Doctor, IDI-3)*.

#### Use of EDSS

ASHAs screened 22,046 adults and identified 900 (4.1%) screen positive cases who were referred to the doctors for further diagnosis and treatment. Initially, ASHAs screened only 4 individuals per day and this increased to 8 individuals per day. While the focus of the study was on CMDs, any individual identified at increased risk of CMD was advised to seek care for their mental health condition and provided information about available doctors and mental health professionals. Those with more severe mental disorders or those with severe depression or increased risk of suicide were followed up more intensively by the ASHAs to check if they had visited a clinician, and family members were also involved after seeking the individual’s approval for doing so. As ASHAs became more confident, they started providing support and advice in the form of talk therapy to the more difficult cases too. The doctors initially took around 30 minutes to diagnose and manage each patient using the EDSS based algorithm, but this reduced to about 20 min with practice. Nearly all doctors acknowledged that the intervention helped them to gain more skills in managing CMDs. They felt the EDSS application assisted in monitoring and treating patients through its ability to provide customized information and advice in advance of consultations. 50% of the doctors also expressed that using the EDSS helped reduce paperwork.

*“This program helped us professionally to improve our counselling skills and how to spend 180 more time with patients and how to speak with patients. In this way we are improving our skills as a doctor.” (Doctor, IDI-4)*.

However, there were some doctors who cited technical barriers on using the EDSS application. For example, some words to elicit symptoms were difficult to translate into local language (Telugu) especially while counselling and treating the patient.

*“Signals…very poor at time. Sometimes they [the data] are not synchronizing. So many times, we faced inconvenience because of this synchronizing process. The data collected by ASHA’s did not synchronize into our tabs. It took so many days for that [to be sorted out].” (Doctor, IDI-4)*.

#### Use of IVRS

There were separate IVRS messages for individuals, ASHAs and primary care doctors. Overall, 14,849 IVRS calls were placed over a period of 10 months (13,400 calls to screen positive individuals with a success rate of 54.2%; 1449 calls were to ASHAs and doctors with a success rate of 56%. Success was defined as the number of calls that were received or heard either completely or partially. Few participants were aware of the voice messages sent through IVRS. Most believed these were not useful for the community members. Reasons provided included: people do not listen to messages unless forced to; non-interactive messages were not useful; single phone ownership in the household was limited, particularly when men in the family controlled mobile phone use; and illiterate individuals did not recognize numbers and considered them as spam.

*“One of the community members shared, “No, I don’t have any idea about messages. I never checked whether I got it or not. I saw few messages but do not know what messages they are… I never paid much interest.” (Community member, FGD 3)*.

### Population characteristics-predisposing characteristics, enabling factors, needs

Mental health awareness in the community was low before we started the intervention [20] though in our formative work we could gather that there was a need for mental health care and a need to increase knowledge about common mental disorders and reduce stigma [21, 22]. In order to increase mental health awareness and reduce stigma related to help-seeking a large campaign was rolled out. This was done after seeking permission from regional and local authorities, and the local administration provided full support to share such information in their communities by allowing use of school or village playgrounds, putting up campaign posters at key places such as schools, local administration offices and primary health centres, and doing public announcements throughout the villages about the anti-stigma campaign and drama shows.

#### Mental health awareness and perceptions about stigma

Almost all participants, irrespective of gender and education, recognized that stigma is associated with mental illness, and was highly prevalent. Village leaders revealed that labelling and rejection in all areas of life were common manifestations of stigma. Patients specifically mentioned that they were afraid of expressing themselves due to fear of losing respect in the community. Similarly, doctors expressed that such labelling within the social context was a complex issue, and played an important role in diagnosis, and the treatment.

*“In my family also, people won’t agree to go to a hospital. Now, I never told them that I am coming here for treatment. They already shouted at me for talking with ASHA workers and for taking doctor appointment.” (Screen positive-visited the doctor, IDI15)*.

FGDs with ASHAs revealed that shame, embarrassment, rejection and subsequent social exclusion were major stigmatizing issues amongst people suffering from mental illness.

*“They [community member] fear that other people [will] think they have mental [health] issues and they are mad. Many people have these perceptions.” (ASHAs, FGD2)*.

Interviews with village leaders and community members demonstrated that almost all participants had limited knowledge of mental illness, regardless of their age, gender and education. Some attributed poor knowledge and understanding to stigma attached to mental illness.

*“They [community members] might be thinking that mental health problems are like a contagious disease.” (Community member, FGD-4)*.

Screen positive individuals, who visited the doctor, indicated that their family, friends, and neighbours did not take mental health seriously in spite of acknowledging it as an issue. As one participant mentioned:

*“…..sometimes we tell our problems to friends and family members but normally people would not consider the problem as a serious one” (Community member, FGD-11)*.

One third of community members tended to associate improvement of mental health with provision of medicine. Two doctors expressed that people who came for treatment gave constant importance to the need for medicines and expressed dissatisfaction when no medicines were prescribed. The majority of the FGD participants shared that in most cases, family members disapprove visiting a psychiatrist - especially where a woman is suffering from CMDs. Two community leaders mentioned that in some families, the elders would not allow daughters or daughters-in laws to seek treatment, especially for mental illness. They further added that many middle-aged women (between 35 and 40 years old) had CMDs because of several stressors at home such as alcoholism among husbands, financial crises, delay in getting their daughter married. One village leader reported that,

*“I think women have less freedom to visit doctors whereas men don’t need permission. They are free to go anywhere, anytime; whereas it is not like that they have to inform family members.” (Village leader, IDI- 6)*.

Almost all community members, village leaders, and doctors recommended the need to educate communities about different mental illnesses. They asserted that such education would increase knowledge and help reduce stigma. Almost all participants suggested that the role of long-term use of dramas or street plays, and videos to sensitize communities about mental health issues.

#### Delivering the mental health awareness and anti-stigma campaign

Two types of brochures and pamphlets were distributed 2–3 times for nearly 3 months through door-to-door campaign in each village to raise mental health awareness. In addition, two types of pictorial posters with messages on mental health issues were developed to create awareness about mental health. A promotional video about the programme and a video of the patient sharing his lived experience was screened at least twice in each village, and it was also shown during the door-to-door campaigns. A drama highlighting mental health stressors, its effect on mental disorders, and need for seeking care was implemented by a local theatre company. Live shows of the drama were staged in 5 villages, video of the show was screened in the others. On an average, the total duration of these live performances or screening of the videos was around 90 minutes. The duration for screening of video clipping of the drama and the other two videos done during the door-to-door campaigns was 30 minutes. While the door-to-door campaign shared different components of the mental health awareness campaign to each household and discussed them in small groups, the live drama shows were attended by 70–300 community members. Video clippings of the live show were also screened later and were watched by about 30–50 community members per village. No attendance was taken but a headcount based on observation was used to estimate the numbers.

#### Accessing health care: distance, financial and socio-cultural constraints

 The distance from home to health facility was a challenge for many participants. Almost all participants affirmed that it took more than an hour to reach the healthcare centre. Most community members including village leaders felt that the access to PHCs was difficult. Particularly for the poor and the daily wage earners, traveling time and cost become critical barrier to seeking care. Some of them further explained that public transport was poor, and private transport was expensive.

*“With transportation issues, some people are not going… We need to walk 3kms to come out of the village, from where they have to go to another village and walk another 1km to reach PHC, so because of these transportation issues some people are not going.” (ASHA, FGD-5)*.

IDIs with village leaders revealed that people having mental health problems generally could not express their problems to their neighbours. However, if available, they are willing to share their problems with doctors, but to do so they needed support to access health services Almost all doctors shared their concern about low follow up and felt that as soon as people start feeling better after their first visit, they tend to stop treatment. Domestic violence, family restrictions, preoccupations with work were the other reasons which prevented doctor visits, as highlighted by the village leaders. About one-half of ASHAs indicated social barriers to care delivery, describing their reluctance to enter some houses which either belonged to economically well off people, or to higher castes.

#### Organizing health camps to increase access to care

Altogether 104 health camps were organized in villages to facilitate easier access to doctors and treatment. Out of the 731 individuals who sought care from the primary care doctors, 716 sought care in the health camps, with the remainder using services at the primary health centre. All participants valued health camps in their locality. Health camps were perceived as good for sensitization with benefits for accessibility for treatment. Most village leaders indicated that individuals would not have sought care for their mental health problems if they had to travel.

*“Definitely these [health] camps were useful. If doctors come voluntarily, check everyone and provide treatment for the patients…it is really good.” (Community member, FGD 10)*.

ASHAs, doctors and some screen positive individuals indicated that health camps also provided an opportunity for patients to discuss problems amongst themselves, and seek advice from each other, based on personal experiences and coping skills.

*“When we used to hear their problems, we felt that our problems are very less and we tried to handle our problems and helped them also to come out of them.” (ASHA FGD)*.

### Health behaviour: personal health practices, use of health services

 The qualitative data provided details about both personal health practices and factors that were perceived by the community as critical for determining increased mental health service use. The backend analytics provided additional information about the quality of care provided and whether it was based as per the algorithm.

#### Mental health service use

ASHAs were able to follow up 888 (98.7%) of all screen positive cases during the one-year intervention period and more than 80% of screen-positive individuals sought care from the primary care doctors. Of those who sought care from the doctors, 514 were females. Of the 242 who received a clinical diagnosis, about 50% suffered from emotional stress, mild/moderate depression or suicide risk. The remaining could not be given a criteria-based diagnosis though they had been screened positive by the ASHAs. It could also be that some natural remission may have occurred by the time the individuals sought care from the doctors. More details of the screening, diagnosis, followup, reasons for differences in clinical diagnosis and screen positivity have been outlined in the earlier paper that outlined all the quantitative results from the project [23]. Some of the participants suggested that health camps should be held on holidays to enable maximum participation. They also provided advice on ideal scheduling, with communication of this through local cable networks. Half of the doctors, ASHAs and the field staff suggested the need to conduct additional training to continuously update knowledge.

*“the training should be provided for at least entire one day at regular intervals and 324 follow up classes should be conducted at least once in a month” (Doctor,IDI-1)*.

#### Preference for awareness campaign strategy

Most participants were aware of most of the anti-stigma campaign activities with the door-to door campaign and drama considered the most popular strategies. Village leaders and ASHAs were enthusiastic about culturally-tailored dramas and felt that these were valuable for both literate and illiterate community members.

*“The program was very good…90% useful. One drama was shown to us as a part of the program. It was good, and people understood how a person suffers from mental disorders. We all felt very happy and people changed a lot.” (Village leader, IDI- 8)*.

For community members, the door-to-door campaign seemed to be the most preferred way to increase awareness about mental health, as one of them said:

*“Door to door campaigning is better because everyone get[s] information and knowledge about mental health.” (Community members, IDI-13)*.

About 50% of the village leaders expressed that the door-to-door visit was more beneficial for less active community members and for those who were difficult to approach or contact. Community members preferred watching dramas and videos of the dramas. Half of the community members suggested to conduct monthly health meetings at regular intervals, to increase awareness and minimize stigma related to help-seeking.

*“It is very important to conduct review meetings…every fortnight or month. So that we can get an exact idea about the status of the patients and make our services [mental health services] better.” (Doctor, IDI- 5)*.

#### IVRS use

Few were in favour of pre-recorded voice messages sent through IVRS, as they felt this would have limited reach to those familiar with technology. One third of participants favoured the idea of receiving information through pamphlets.

#### Social support

More than half of the community members, village leaders suggested that more social support was required at both family and the societal levels, such as support groups and self-help techniques to help families to deal with mental disorders. Some thought that the intervention could be improved if implemented through PHCs in collaboration with the established rural ambulance system. Few others suggested that patients should be given complete support in terms of logistics, follow-ups and referrals, till completion of the treatment.

### Outcomes: perceived health status, evaluated health status, consumer satisfaction

Different stakeholders expressed their views about the intervention during the qualitative interactions. Quantitative data from this project showed that among those in need of mental health services for CMDs, there was an increase in mental health services use from 3.3 to 81.2% following the intervention, and an improvement in depression and anxiety scores [23].

#### Appraisal of the SMART Mental Health programme

Most participants held favourable views about the interventions, particularly in enhancing knowledge and understanding about CMDs and steps to promote mental well-being. Some participants felt that the interventions were more useful for older people, and women as a perceived greater need for mental health care in these demographics. Community members who were seeking treatment felt that the programme provided them an opportunity to share their problems with someone like ASHAs. Most community members expressed a desire for the intervention to continue beyond the research period.

*“We always welcome your health programmes. These programmes should be continued forever. People shall always be in need of a psychiatrist because some (or) other shall always face some problem regularly and they need someone to lift them up.” (Community leader, IDI-9)*.

The government official when interviewed, suggested extending the SMART mental health programme under an umbrella of public private partnership for the benefit of larger populations, and to make it more sustainable.

*“The team can support in a PPP programme which is a public private partnership by explaining the SMART Health programme and its objective.” (Government Official IDI 1)*.

#### Benefits of involving ASHAs

Nearly all participants felt that involvement of ASHAs in the study was positive as individuals were more likely to be comfortable sharing their personal problems with ASHAs. Two-thirds of community members expressed that ASHAs not only paid repeated visits to enquire about their 384 health, but also tried to motivate them to visit the doctor for treatment, and this was appreciated.

*“Yes, definitely they [community members] pay attention to ASHA. Ladies mostly approach ASHA only whatever be their problem. It is only the ASHA who takes care of ladies all through their pregnancy. Once the child is delivered, they take care of vaccines and other things.” (Community Member, FGD-1)*.

All village heads and community leaders believed that ASHAs were effective in building rapport with people in villages and spreading information. Secondly, ASHAs, being native to the community, did have better understanding about the local issues.

*“ASHA workers are really good. They persuade us to go to doctor and sometimes they accompanied us also to visit the hospital.” (Community member, FGD-4)*.

Doctors explained that ASHAs’ rapport with the community not only helped them to play a major role in implementing the intervention but also encouraged them in administering the treatment and ensuring follow up. Screen-positives individuals, who visited the doctor, felt that ASHAs inquiring about peoples’ health provided them comfort. ASHAs sometimes persuaded them to visit the doctor and sometimes they even accompanied them.

#### Appraisal about the anti-stigma campaign and mental health services

Most community members and leaders indicated that the intervention increased their knowledge of CMDs which, in turn, raised its profile as a health issue. This increased the acceptability of seeking treatment. Many village leaders agreed that the intervention helped individuals by making resources readily available without additional financial burden. Nearly half of the doctors highlighted that the intervention increased community understanding about stress, depression, suicidal risk, and helped individuals identify symptoms of stress. Two doctors indicated that the project helped them gain knowledge about different ways of treating CMDs.

*“Interacting with the patient and getting information about their health is really good. Actually, these people don’t know that they have CMD they imagine it differently …as mad …as abnormal.” (Doctor, IDI 03)*.

## Discussion

The results of the current process evaluation provided information on intervention implementation. Overall, the process evaluation demonstrated that the interventions were feasible and acceptable by different stakeholders. The interventions were perceived as being valuable in enhancing participants’ understanding of CMDs as well as promoting their mental well-being. Our earlier study [11] and this process evaluation demonstrated that the use of the Andersen’s Model of Health Service Use provided a theoretical framework to identify several facilitators/barriers and provides greater contextual information for an implementation intervention. The three key elements of the intervention were task-sharing with primary health workers and training primary health workers; enabling primary health systems based mental health services using EDSS; and conducting a community-based campaign to raise mental health awareness and reduce stigma related to help-seeking.

### Task sharing the training of primary health workers

Task sharing and training primary care workers in providing care for a number of health disciplines including mental health have been used in many countries [24]. Task-sharing was facilitated in this intervention by training the existing primary health workers—ASHAs, and primary care doctors-to screen, diagnose and manage individuals with CMDs. Task sharing and providing care using primary health workers is outlined in the National Mental Health Policy and Mental Health Action Plan 2013–2020 [25, 26]. The ability of ASHAs and the primary care doctors to screen more than 22,000 individuals and then follow-up with almost all screen positive individuals indicated that the overall premise of task sharing was successful. The community leaders and other stakeholders were also appreciative of the intervention and especially the beneficial role of ASHAs was pointed out both by the community members and doctors. Using lay health workers to deliver mental health care has been used by other researchers too [27], but in this project we have avoided using any additional resources such as counsellors to enhance the service delivery as no such resource currently exists within the public health system. While adding such resources are beneficial, the scalability of such an approach is debatable. Though, the government has started the process of counsellors for non-communicable disorders, but as yet no such resource is available for mental disorders which need more specific skills.

### Enabling mental health service delivery

The EDSS was used as an enabler to make the mental health service delivery more efficient. This was coupled with some other measures such as organizing health camps to increase access to care and using IVRS to support community members, ASHAs and primary care doctors to make the system more responsive. Overall, the use of mental health services by those needing such care increased significantly over the intervention period [23]. The use of EDSS was found to reduce paperwork and make the system more efficient and also helped improve the counselling skills of the doctors as it guided them about simple questions in the form of talk therapy that could be asked for each individual. The community also felt that the ASHAs checking on their wellbeing and requesting them to seek care was helpful and this was also due to the prioritization of the follow-up process that was enabled by the EDSS. This provides justification to earlier research on use of EDSS in clinical practice [28–31] and allowed to generate evidence about how to deliver such EDSS enabled mental health care in resource poor settings. However, there were also some key learnings that one needs to factor in while developing these – quality of internet connectivity, penetration of smart phones in the community (which can increase the level of sophistication of any EDSS enabled care), and overall acceptance amongst the community and primary health workers of such methods of delivering care. While as part of our continuous processes of improving the system we had developed some strategies to enable hotspots at the primary health centres, identify key areas within the villages which had better mobile network signals, and liaise with the primary health workers to trouble-shoot any issues that they faced while delivering the intervention during our routine monitoring, issues did appear at times throughout the course of the intervention which were taken care off [32]. IVRS was used to increase the ability of both the community members and primary health workers to eb more responsive and manage the mental health needs. However, lack of phones with every individual, especially women, and failure of the community members to realize the source of the call as it often came from an unidentified number, reduced the ability of IVRS to bring about change in service delivery or facilitate its delivery. This was captured through the qualitative interviews, even though the messages were sent out in adequate numbers. Future research will need to focus on improving the mechanisms of delivery of IVRS and make it more effective. The benefits of technology-enabled mental health service delivery models have been reported in recent reviews [33] and is recommended as a critical strategy for scaling up mental health care by policy makers [26]. Inability to pay for public transport and distances to the primary health centres were often cited as causes for not accessing care. Some of these factors were similar to earlier research [32]. One strategy used to increase access was organization of health camps where those needing mental health care to visit the primary care doctors. This process was supported by ASHAs and monitored by the implementation team. The health camps per se were found to be beneficial by participants and village leaders, and more such camps were sought. Almost 98% of those who sought mental health care did so at the health camps, and it underlines the value of organizing such health camps for delivery of health care in villages. Increasing access is a critical component of the Universal Health Care programme of the Government of India – Ayushman Bharat – and many states have been using health camps to increase the each of health care in the more hard-to-reach areas, including hilly terrains. The premise of establishing Health and Wellness Centres in rural areas as part of that programme is also aligned with increasing access. Mental health is being considered as an important and neglected health condition that should be managed at the Health and Wellness Centres. Once the Health and Wellness Centres are more established it will become easier to increase delivery of care using these centres where health camps could be better integrated in a systematic manner.

### Mental health awareness campaign

The community had a felt need for increasing mental health awareness and the formative work had identified stigma as one of the barriers for seeking help [21]. The pre-post evaluation of the anti-stigma campaign showed the beneficial effects of the campaign [20] and those positive effects continued to be present even after 2 years [34]. The limited knowledge of CMDs among participants prior to receiving the intervention seen in this study correlates well with previous literature [35, 36]. Community members identified various barriers in care-seeking. Stigma was perceived as a barrier to seeking mental health care and the mental health awareness campaign was perceived as beneficial to reduce stigma and increase mental health awareness. Women were also disadvantaged as due to stigma they were often dissuaded from receiving mental health care even if they needed it. The effect on stigma on gender has been discussed earlier and it has been shown that women are more likely to seek help for mental health [37], but it is also known that if enabling, predisposing and need level factors are controlled for, men are more likely to seek care if severely unwell [38]. The socio-cultural factors affecting women’s ability to seek mental health care need further exploration. This evaluation clearly demonstrate that the limited knowledge of mental health was associated with social stigma, labelling and pre-occupation with negative psychological feelings (i.e. depression, low self-esteem, mood disorders), all of which have been linked with lower treatment adherence [39]. The common stigmatizing perceptions expressed were similar to earlier research [40]. The door-to-door campaign and drama shows were specifically alluded to by the community members as beneficial to gain knowledge about mental health. Theatre as a means of sharing mental health knowledge had been used but with limited success beyond immediate effect [41]. However, we had found that the stigma campaign had longer term benefits [34], and the drama was identified as a key method for delivering the campaign in discussion with the community both in our earlier assessment [20] and in this evaluation. Social contact is the process where individuals with mental disorders share their experiences either through live or video talks with an aim to reduce stigma related to mental health. It has been identified as the most important community-based intervention that has been effective in delivery of an anti-stigma campaign [42]. The use of drama/theatre is considered as a form of indirect ‘social contact’ by which the community gains knowledge about mental health outcomes and stigma by observing scenes around mental disorder and stigma. This has implications for larger anti-stigma campaigns which traditionally depend on brochures and pamphlets, a strategy that is less effective unless supported through intensive door-to-door campaigns or use of innovative strategies such as drams, street theatres, and social contact related videos. Changes in awareness of community can encourage and support communities to influence community leaders to build more enabling environment for treating individuals suffering from mental disorders.

### Impact for future research and policy

Many socio-economic characteristics play an important role in utilization of health services. Andersen’s model not only allowed us to ascertain the facilitators and barriers related to our intervention, but the theoretical framework also allowed us to postulate how some of the stigma and service delivery outcomes that we observed [20, 23, 34] could have been affected by environmental factors, personal characteristics, and health behaviours of the communities. These factors affect their decisions to seek care, and the outcomes in turn affect their long-term decisions and development of a sustainable system of care. While in this evaluation we could not assess how the observed and perceived benefits affected long term mental health service delivery in the communities, it did provide some evidence about the perceived benefits of the intervention for different stakeholders. Village leaders and policy makers were keen to see the intervention grow. Future research could focus on long term effect of these kind of interventions and also cost-effectiveness. Training of primary health workers on mental health care and on navigation of the applications was a key element. Future research could explore innovative ways to train such health workers using online platforms and factor in booster sessions. The evaluation also showed some other areas that could be enhanced through future research. Access to a mobile phone among community members was necessary to make the IVRS more effective, hence future research should take steps to ensure these messages are delivered more efficiently. Future applications should be supported by platforms that are easier to modify, so that monitoring could be reduced. Health camps were a key component of the service delivery and future research should explore how health camps could be better integrated within the existing health system with an ability to integrate it with the Health and Wellness Centre activities. Through this intervention a multimedia approach was used to enhance mental health knowledge and some elements were identified as most beneficial by the stakeholders. Future research could tease out the different components to ascertain which interventions were most effective. From a policy perspective, the programme was found to be acceptable, feasible and led to beneficial outcomes. Though the pre-post design of the study did not allow more robust results, it gave us vital information about the complex intervention and this evaluation allowed us to understand different barriers and facilitators. The delivery of the anti-stigma campaign and technology-enabled mental health services delivery model were found to be beneficial with potential for scale up. The different components of the intervention could be broken down to smaller components and adapted to local needs. Subsequent steps would be to conduct a more robust randomized controlled study which assessed cost-effectiveness and involved translational research components which were appropriately evaluated using implementation science framework.

## Conclusions

In conclusion, this process evaluation complements the results ascertained through different quantitative assessment and as was reported earlier. The Andersen’s theoretical framework provided a structure and context to understand how different factors could have functioned synergistically to help in delivery of the intervention as was observed in this project. It also provides a better understanding of the processes that could be enhanced to make the SMART Mental Health Programme scalable through future research.

## Supplementary Information

**Additional file 1.** FGD and IDI guidelines.

**Additional file 2.** Consolidated criteria for reporting qualitative studies (COREQ) - 32 item checklist.

**Additional file 3.** Key themes and reflective quotes.

## Data Availability

The datasets used and/or analyzed during the current study are available from the corresponding author on reasonable request.
