# Supplementary material for: SMART Mental Health Project: process evaluation to understand the barriers and facilitators for implementation of multifaceted intervention in rural India
Source: Int J Ment Health Syst. 2021 Feb 8;15:15. doi: 10.1186/s13033-021-00438-2 (PMC7871593; doi:10.1186/s13033-021-00438-2)
Supplement: Supplementary file 1 — Additional file 1. FGD and IDI guidelines. [file 13033_2021_438_MOESM1_ESM.docx]

**Additional File 1: FGD and IDI Guidelines**

| **FGD Guide for ASHAs** |
| --- |
| - Please tell us about your experience with Smart Mental Health Programme conducted by George Institute Hyderabad. *Probe:* - *Role in programme,* - *Interaction with project staff, community members and Doctors*   **Training**   - How was your experience in attending the training for implementing the SMART mental activities? *Probe:* - *How useful or not useful was it?* - *How it can be improved?* - *Any other aspects and issues that you felt needed more discussion?*   **Implementation, Screening and Camps**   - What was your experience when you visited the households for asking the questions/ screening? Please give details .*Probe:* - *Were you comfortable in asking those questions? Please state the reasons.* - *Was it useful? If yes, how? If no, please elaborate what was not useful?* - *Do you think ASHAs are the best person to ask these questions? If yes, why? If no, please suggest who will be the best person to ask these questions and why?* - Please tell us your experience in conducting / organizing Health camps in communities? - *Do you think that conducting camps was an effective strategy? If yes. Why?* - *What were some key issues that prevent people from accessing care and follow up?* - *What challenges you faced for motivating people to go for treatment?* - Please tell us your experience with using mhGAP tool? What were the benefits or problems in using the tool? *Probe:* - *Handling the tablet* - *Filling up the information* - *Length, content and language of the questionnaire* - *Any suggestions or lessons learned* - Please tell us about your experience with the IVR messages and how community members and you perceived this experience*. Probe:* - *Implementation, content and language of the message* - *Any suggestions or lessons learned* - What was the level of support you received from GI staff, community members and doctors? Please share your positive as well as negative experiences *Probe:* - *Was this support useful?* - *How can it be improved?*   **Suggestions**   - Overall, what are some of the challenges or difficulties that you are encountering with the program? Please give details. - Please give your suggestions to improve the programme in future? |
| **FGD Guide for Field Investigators & Project Staff** |
| - Please tell us about your experience with Smart Mental Health Programme conducted by George Institute Hyderabad. *Probe:* - *Role in programme,* - *Interaction with project staff, ASHAs, and community members and Doctors*   **Training**   - How was your experience in attending the training for implementing the SMART mental activities? *Probe:* - *How useful or not useful was it?* - *How it can be improved?* - *Any other aspects and issues that you felt needed more discussion?*   **Implementation**   - Please tell us about your experience in implementing the activities of anti-stigma campaign. - *Tell us about your experience with the community members, their participation or non-participation for activities of campaign (probe for each activity separately)* - *Any suggestions or lessons learned.* - Please tell us your experience in conducting / organizing Health camps in communities? - *Do you think that conducting camps was an effective strategy? If yes. Why?* - *What were some key issues that prevent people from accessing care and follow up?* - *What challenges you faced for motivating people to go for treatment?* - Please tell us about your experience with the IVR messages and how community members and you perceived this experience. *Probe:* - *Implementation, content and language of the message* - *Any suggestions or lessons learned* - Please tell us your experience with using mhGAP tool? What were the benefits or problems in using the tool? *Probe:* - *Implementation, Length, content and language of the questionnaire* - *Any suggestions or lessons learned* - What was the level of support you received from GI staff, ASHAs , community members and Doctors ? *Probe* - *Was this support useful?* - *How can it be improved?* - What was your experience in working with ASHAs? Please share your positive as well as negative experience. *Probe:* - *Interaction with people* - *Handling the tablet* - *Motivate people to visit doctor* - *Any other* - What was your experience in working with doctors at PHCs/camps? Please share your positive as well as negative experiences Probe: - *Interaction with people* - *Handling the tablet;* - *Filling up the information* - *Motivate people to take treatment* - *Any other*   **Suggestions**   - Overall, what are some of the challenges or difficulties that you are encountering with the program? Please give details. - Please give your suggestions to improve the programme in future? |
| **FGD Guide for community members (screen negatives)** |
| - Please tell us about yourself in brief? (*Probe: Name (optional), village, age, literacy status, economic status, type of family (nuclear / extended), occupation, marital status, ownership of mobile phone, distance from the hospital***.)** - Give us your opinion about the Smart Mental Health Programme conducted by George Institute, Hyderabad in your community. - Can you recall any of the activities (Probe: *Anti- stigma campaign, ASHAs visit, screening, IVR messages and Health camps*). - Did you attend /participated in any of the activities. (probe for each activity) - Did you share the information with your family, friends, colleagues or neighbors? If yes, what was their opinion? If no, what were the reasons?   **Anti-stigma campaign**   - Are you aware of the anti-stigma/mental health awareness campaign? - Was it useful? If yes, how? (Probe: what components were useful to encourage service use) .If no, please elaborate what was not useful? - Do you have any suggestions for improvement?   **Screening & Camps**   - What was your experience when ASHAs visited households for asking the questions/ screening? Please give details - Were you comfortable in talking with ASHAs? - Was it useful? If yes, how? If no, please elaborate what was not useful? - What can be improved? - Do you think ASHAs are the best person to ask these questions? If yes, why? If no, please suggest who will be the best person to ask these questions - Are you aware of Health camps organized by George Institute in your community? If yes, how did you learn about these camps? - *Do you think that conducting camps was an effective strategy? If yes. Why?*   **Suggestions**   - Do you think this programme has any effect- positive/negative on the community? Please give your suggestions to improve the programme in future? - Is there any new initiative taken by the community members to support people to receive treatment? |
| **FGD Guide for Patients (not visited the doctor)** |
| - Please tell us about yourself in brief? (*Probe: Name (optional), village, age, literacy status, economic status, type of family (nuclear / extended), occupation, marital status, ownership of mobile phone, distance from the hospital*.) - Give us your opinion about the Smart Mental Health Programme conducted by George Institute, Hyderabad in your community. - Can you recall any of the activities (Probe: *Anti- stigma campaign, ASHAs visit, screening, IVR messages and Health camps*). - *Did you attend /participated in any of the activities. (probe for each activity)* - *Did you share the information with your family, friends, colleagues or neighbours? If yes, what was their opinion? If no, what were the reasons?*   **Anti-stigma campaign**   - Are you aware of the anti-stigma/mental health awareness campaign? - *Was it useful? If yes, how? (Probe: what components were useful to encourage service use). If no, please elaborate what was not useful?* - *Do you have any suggestions for improvement?*   **Screening & Camps**   - What was your experience when ASHAs visited households for asking the questions/ screening? Please give details - *Were you comfortable in talking with ASHAs?* - *Was it useful? If yes, how? If no, please elaborate what was not useful?* - *What can be improved?* - *Do you think ASHAs are the best person to ask these questions? If yes, why? If no, please suggest who will be the best person to ask these questions* - Are you aware of Health camps organized by George Institute in your community? If yes, how did you learn about these camps? - *Were these camps easy to access?* - *Were doctors easy to approach and consult?* - *Whether these camps should be organized in future also? If yes, please give reasons.*   **App/IVR**   - Have you received any IVR message? If yes, how was your experience in receiving the IVR messages? Probe*:* - *Was the information useful? If yes, in what ways?* - *Did you find any difficulty in understanding them? Probe : language and content of the message* - *Would you like to receive similar messages in future? If yes, why?? If no, please give reasons?* - *If you have not received this please share the reason for not receiving it?*   **Treatment**   - In your opinion what are the various factors that encouraged and discouraged individuals in seeking treatment? *Probe: age groups, gender and socio, economic status, lack of facility, lack of support, doctors experience, any other* - Please tell me about your experience of managing your illness? Did you faced any difficulty in getting support from *family members/friends/relatives/community members/ASHAs/ doctors/any other*) - In your opinion what are some of the challenges or difficulties that you are facing in getting the treatment? Please give details. *Probe: stigma related factors- stigma of accessing services; people knowing, child of marriageable age; loss of social status, etc*   **Suggestions**   - Do you think this programme must be continued, modified, or discontinued? State your reasons. - Is there any new initiative taken by the community members to support people to receive treatment |
| **FGD Guide for Patients (visited the doctor)** |
| - Please tell us about yourself in brief? (Probe: Name (optional), village, age, literacy status, economic status, type of family (nuclear / extended), occupation, marital status, ownership of mobile phone, and distance from the hospital.) - Give us your opinion about the Smart Mental Health Programme conducted by George Institute, Hyderabad in your community. - *Can you recall any of the activities (Probe: Anti- stigma campaign, ASHAs visit, screening, IVR messages and Health camps).* - *Did you attend /participated in any of the activities. (probe for each activity)* - *Did you share the information with your family, friends, colleagues or neighbors? If yes, what was their opinion? If no, what were the reasons?*   **Anti-stigma campaign**   - Are you aware of the anti-stigma/mental health awareness campaign? - *Was it useful? If yes, how? (Probe: what components were useful to encourage service use). If no, please elaborate what was not useful?* - *How could it be improved?*   **Screening & Camps**   - What was your experience when ASHAs visited households for asking the questions/ screening? Please give details - *Were you comfortable in talking with ASHAs?* - *Was it useful? If yes, how? If no, please elaborate what was not useful?* - *What can be improved?* - Do you think ASHAs are the best person to ask these questions? If yes, why? If no, please suggest who will be the best person to ask these questions - What kind of support did you get from the ASHAs and George Institute staff to visit the PHCc/camp? (probe about ASHA follow up) - *Was it useful or not?? If yes, how? If no, please elaborate what was not useful?* - *What can be improved?* - Are you aware of Health camps organized by George Institute in your community? If yes, how did you learn about these camps? - *How was your experience at the Health camps? Probe:(Registration related, who attended you first, waiting period, any other).* - *Were these camps easy to access?* - *Were doctors easy to approach and consult?* - *Were you comfortable in talking to doctors?* - *Would you like to receive such services in future also? If yes, please give reasons.*   **Treatment received**   - What kind of treatment did you receive from the doctor? – Medicines/counselling/referral? - Were you able to continue treatment as advised and for how long? - Was there any problem in continuing treatment? If yes, what were they? *Probe: stigma related factors- stigma of accessing services; people knowing, child of marriageable age; loss of social status, etc* Did the ASHAs follow up with you? Was it helpful? If yes, how? If no, why? - In your opinion what are some of the challenges or difficulties that you are facing in getting the treatment? Please give details.   **IVR messages**   - Are you aware of the IVR messages sent by George Institute related with this programme? Please tell us about your experience in detail? - Sending such type of messages is a good strategy? Please comment. - In your opinion what kind of information is helpful to patients /family members to receive treatment?   **Suggestions**   - Do you think this programme has any effect- positive/negative on the community? Please give your suggestions to improve the programme in future? - Is there any new initiative taken by the community members to support people to receive treatment? |
| **Interview Questions for Doctors** |
| - Name (optional)………………. - Age (yrs)………………..   **Training**   - Please tell us about your experience with Smart Mental Health Programme conducted by George Institute Hyderabad. *Probe: Role in programme, interaction with project staff, ASHAs, and community members* - How was your experience with training programme conducted by George Institute? *Probe: How useful or not useful was it, How it helped you professionally and How it can be improved?,What were the aspects and issues that you felt needed more discussion? And suggestions for future training programme****?***   **App/IVR**   - How was your experience in using the app/tablet provided by the George as part of project activity? *Probe: Was training helpful in handling apps and tabs? Level of support received from GI staff to handle app/tablet and how? positive and negatives? And how do we improve this support?* - Did you receive IVR messages? If yes, how was your experience in receiving the IVR messages*? Probe: about positive and negative experiences? And how do we improve this?* If no, then please share the reason for not receiving it. - In your opinion , what were the issues related to the questionnaire ?*Probe: Length, content and language of the questionnaire* - Implementation - In your opinion what are the reasons for follow up patients revisiting or not visiting the PHC for continuing treatment? - What was the level of support you received from GI staff? *Probe: Do you think this support was useful; how can it be improved?* - In your opinion what were some key issues that prevent people from accessing care and follow up. *Probe: Do you think that conducting camps were effective strategy*   **Suggestions**   - Please give your suggestions to improve the programme in future interventions? |
| **Interview questions for Government Officials** |
| - Name (optional): ………………………….……….. - Designation: ………………………….……….. - Name of the department/organization: ……..……………………………………………… - Please tell us about yourself in brief? ( activities of department, your role etc.) - What are the specific initiatives/interventions being undertaken by your ministry/ organization in the area of mental health? - Give us your opinion about the SMART Mental Health Programme conducted by George Institute, Hyderabad in your community. *Probe: What were the positive?* What were the negatives and How could the programme be improved? - Could this be a model that would be beneficial for the government? What are beneficial and what are the not beneficial components? Probe. - In what ways could the government be part off and facilitate the process of integrating components identified above as beneficial into the current health system? |
| **Interview Questions for Community Leaders** |
| - Please tell us about yourself in brief? (*Probe: Name, age, gender, literacy status, economic status, type of family (nuclear / extended), occupation, number and gender of children, ownership of mobile phone, distance from the hospital/health facility)* - Give us your opinion about the Smart Mental Health Programme conducted by George Institute, Hyderabad in your community. - Can you recall any of the activities (*Probe: Anti stigma campaign, ASHAs visit, screening, IVR messages and Health camps).* - Did you attend /participated in any of the activities. (*Probe for each activity*) - Did you share the information with your family, friends, colleagues or neighbors? If yes, what was their opinion? If no, what were the reasons? - In your opinion what are the various factors that encouraged and discouraged individuals in seeking treatment? (Probe: age groups, gender and socio, economic status)   **Anti-stigma campaign**   - Were you aware of the anti-stigma/mental health awareness campaign? - Was it useful? If yes, how? (Probe: what components were useful) - If no, please elaborate what was not useful? - How could it be improved?   **Screening & Camps**   - What was your experience when ASHAs visited households for asking the questions/ screening? Please give details - Were you comfortable in talking with ASHAs? - Was it useful? If yes, how? If no, please elaborate what was not useful? - What can be improved? - Do you think ASHAs are the best person to ask these questions? If yes, why? If no, please suggest who will be the best person to ask these questions - Please share your experience about visiting the doctor at the PHC? - Was it useful? If yes, how? If no, please elaborate what was not useful? - What can be improved? - What was the support did people receive from the ASHAs and George Institute staff to visit the PHC? - Was it useful or not?? If yes, how? If no, please elaborate what was not useful? - What can be improved? - In your opinion what are the reasons for follow up patients visiting or not visiting the PHC for continuing treatment? - Are you aware of Health camps organized by George Institute in your community? If yes, please share your experience? - How did you learn about these camps? - Were these camps easy to access? - Were doctors easy to approach and consult? - Would you like to receive such services in future also? If yes, why   **IVR messages**   - Are you aware of the IVR messages sent by George Institute related with this programme/camp. Please tell us about your experience in detail? - Sending such type of messages is a good strategy? Please comment. - In your opinion what kind of information is helpful to patients /family members to receive treatment?   **Suggestions**   - What were the positive or negative effects of the overall programme on the community /you / your family members? - Do you think it must be continued, modified, or discontinued? State your reasons. - Is there any new initiative taken by the community members to support people to receive treatment? Please give your suggestions to improve the programme in future? |
| **Interview questions for patients (given medical /psychological treatment)** |
| - Please tell us about yourself in brief? (*Probe: Name (optional), village, age, literacy status, economic status, type of family (nuclear / extended), occupation, marital status, ownership of mobile phone, distance from the hospital*.) - Give us your opinion about the Smart Mental Health Programme conducted by George Institute, Hyderabad in your community. - Can you recall any of the activities (Probe: *Anti- stigma campaign,ASHAs visit, screening, IVR messages and Health camps*). - Did you attend /participated in any of the activities. (probe for each activity) - Did you share the information with your family, friends, colleagues or neighbors? If yes, what was their opinion? If no, what were the reasons? - In your opinion what are the various factors that encouraged and discouraged individuals in seeking treatment?  *Probe: age groups, gender and socio, economic status*   **Screening & Camps**   - What was your experience when ASHAs visited households for asking the questions/ screening? Please give details   - Were you comfortable in talking with ASHAs?   - Was it useful? If yes, how? If no, please elaborate what was not useful?   - What can be improved? - Do you think ASHAs are the best person to ask these questions? If yes, why? If no, please suggest who will be the best person to ask these questions - What kind of support did you get from the ASHAs and George Institute staff to visit the PHC?   - Was it useful or not?? If yes, how? If no, please elaborate what was not useful?   - What can be improved? - Are you aware of Health camps organized by George Institute in your community? If yes, how did you learn about these camps? - How was your experience at the Health camps?  *Registration related, who attended you first, waiting period, any other.* - Were these camps easy to access? - Were doctors easy to approach and consult? - Were you comfortable in talking to doctors? - Would you like to receive such services in future also? If yes, please give reasons   **Treatment received**   - What kind of treatment did you receive from the doctor? – Medicines/counselling/referral? - Were you able to continue treatment as advised and for how long? - Was there any problem in continuing treatment? If yes, what were they? - Did the ASHAs followup with you? Was it helpful? If yes, how? If no, why?   **IVR messages**   - Are you aware of the IVR messages sent by George Institute related with this programme/camp? Please tell us about your experience in detail? - Sending such type of messages is a good strategy? Please comment. - In your opinion what kind of information is helpful to patients /family members to receive treatment?   **Suggestions**   - Do you think this programme has any effect- positive/negative on the community? - Is there any new initiative taken by the community members to support people to receive treatment? Please give your suggestions to improve the programme in future? |
| **Interview questions for patients (visited doctors & referred)** |
| - Please tell us about yourself in brief? (*Probe: Name (optional), Village, age, literacy status, economic status, type of family (nuclear / extended), occupation, marital status, ownership of mobile phone, distance from the hospital*.) - Give us your opinion about the Smart Mental Health Programme conducted by George Institute, Hyderabad in your community. - Can you recall any of the activities *(Probe: Anti- stigma campaign, ASHAs visit, screening, IVR messages and Health camps).* - Did you attend /participated in any of the activities. (*Probe for each activity*) - Did you share the information with your family, friends, colleagues or neighbors? If yes, what was their opinion? If no, what were the reasons?   **Screening & Camps**   - What was your experience when ASHAs visited households for asking the questions/ screening? Please give details - Was it useful? If yes, how? If no, please elaborate what was not useful? - Were you comfortable in talking with ASHAs? - What can be improved? - Do you think ASHAs are the best person to ask these questions? If yes, why? If no, please suggest who will be the best person to ask these questions - What kind of support did you get from the ASHAs and George Institute staff to visit the PHC?   - Was it useful or not?? If yes, how? If no, please elaborate what was not useful?   - What can be improved? - Are you aware of Health camps organized by George Institute in your community? If yes, how did you learn about these camps? - How was your experience at the Health camps?  *Registration related, who attended you first, waiting period, any other.* - Were these camps easy to access? - Were doctors easy to approach and consult? - Were you comfortable in talking to doctors? - Would you like to receive such services in future also? If yes, why   **Treatment received**   - What kind of treatment did you receive from the doctor? – Medicines/counselling/referral? - Were you able to continue treatment as advised and for how long? - Was there any problem in continuing treatment? If yes, what were they? - Did the ASHAs followup with you? Was it helpful? If yes, how? If no, why? - Did you follow the doctor’s advice of following with a specialist? If yes, whom did you visit and what was your experience? If no, why not?   **IVR messages**   - Are you aware of the IVR messages sent by George Institute related with this programme/camp? Please tell us about your experience in detail? - Sending such type of messages is a good strategy? Please comment. - In your opinion what kind of information is helpful to patients /family members to receive treatment?   **Suggestions**   - Do you think this programme has any effect- positive/negative on the community? - Is there any new initiative taken by the community members to support people to receive treatment? Please give your suggestions to improve the programme in future? |
