# Supplementary material for: SMART Mental Health Project: process evaluation to understand the barriers and facilitators for implementation of multifaceted intervention in rural India
Source: Int J Ment Health Syst. 2021 Feb 8;15:15. doi: 10.1186/s13033-021-00438-2 (PMC7871593; doi:10.1186/s13033-021-00438-2)
Supplement: Supplementary file 2 — Additional file 2. Consolidated criteria for reporting qualitative studies (COREQ) - 32 item checklist. [file 13033_2021_438_MOESM2_ESM.docx]

**Additional File 2: Consolidated criteria for reporting qualitative studies (COREQ): 32-item checklist**

Developed from:

Tong A, Sainsbury P, Craig J. Consolidated criteria for reporting qualitative research (COREQ):a 32-item checklist for interviews and focus groups. International Journal for Quality in Health Care.2007. Volume19, Number 6:pp.349-357

| No | Item | Guide questions/description | Response |
| --- | --- | --- | --- |
| **Domain 1: Research team and reﬂexivity** | | | |
|  | *Personal Characteristics* |  |  |
|  | Interviewer/facilitator | Which author/s conducted the interview or focus group? | SK and SD (trained by AT)conducted the Interviews and FGDs |
|  | Credentials | What were the researcher’s credentials? E.g. PhD, MD | MSc and PhD |
|  | Occupation | What was their occupation at the time of the study? | Research Fellow & Research Assistant –George Institute for Global Health- India |
|  | Gender | Was the researcher male or female? | Male and Female |
|  | Experience and training | What experience or training did the researcher have? | Facilitators had prior experience of conducting the Interviews and Focus Groups. One author (AT) is a Social scientist with experience in qualitative research provided in-person training in conducting Interviews and Focus Groups and analysis to SK and SD. |
|  | *Relationship with participants* |  |  |
|  | Relationship established | Was a relationship established prior to study commencement? | No relationship with the participants was established before the commencement of the study |
|  | Participant knowledge of the interviewer | What did the participants know about the researcher? e.g. personal goals, reasons for doing the research | Prior to any data collection, we provided all potential participants with an overview of the SMART Mental Health study prior to signing of the informed consent. |
|  | Interviewer characteristics | What characteristics were reported about the interviewer/facilitator? e.g. Bias, assumptions, reasons and interests in the research topic | As per the ground rule of FGDs, the moderators introduced themselves at the beginning of each interview and FGD by name, occupation and the purpose of conducting the discussions. |
| **Domain 2: study design** | | | |
|  | *Theoretical framework* |  |  |
|  | Methodological orientation and Theory | What methodological orientation was stated to underpin the study? e.g. grounded theory, discourse analysis, ethnography, phenomenology, content analysis | We used Grounded Theory to inductively derive the themes. |
|  | *Participant selection* |  |  |
|  | Sampling | How were participants selected? e.g. purposive, convenience, consecutive, snowball | Purposive sampling |
|  | Method of approach | How were participants approached? e.g. face-to-face, telephone, mail, email | Face –to -face |
|  | Sample size | How many participants were in the study? | A total of 141 FGD participants and 25 interview participants |
|  | Non-participation | How many people refused to participate or dropped out? Reasons? | None |
|  | *Setting* |  |  |
|  | Setting of data collection | Where was the data collected? e.g. home, clinic, workplace | The data was collected in the community either at the community centers or in the house of the participants as per their convenience |
|  | Presence of non-participants | Was anyone else present besides the participants and researchers? | We did not allow any non-participant at the time of interview and FGDs. Any non-participants were immediately requested to leave if they entered the room where an interview or focus group took place. |
|  | Description of sample | What are the important characteristics of the sample? e.g. demographic data, date | Demographic data of the participants as they represented the diverse group |
|  | *Data collection* |  |  |
|  | Interview guide | Were questions, prompts, guides provided by the authors? Was it pilot tested? | Yes |
|  | Repeat interviews | Were repeat interviews carried out? If yes, how many? | No |
|  | Audio/visual recording | Did the research use audio or visual recording to collect the data? | Digital audio recordings were made and transcribed verbatim |
|  | Field notes | Were ﬁeld notes made during and/or after the interview or focus group? | Field notes were made during the interview and FGD by the note taker. |
|  | Duration | What was the duration of the interviews or focus group? | Each FGD lasted for 60 minutes and IDI lasted for about 35-40 minutes |
|  | Data saturation | Was data saturation discussed? | Yes |
|  | Transcripts returned | Were transcripts returned to participants for comment and/or correction? | No |
| **Domain 3: analysis and ﬁndings** | | | |
|  | *Data analysis* |  |  |
|  | Number of data coders | How many data coders coded the data? | Two coders (SD & AT) coded the data |
|  | Description of the coding tree | Did authors provide a description of the coding tree? | We have done the non-hierarchical coding and the description is provided in text |
|  | Derivation of themes | Were themes identiﬁed in advance or derived from the data? | Themes were derived from the data and then interpreted as per Andersen’s model |
|  | Software | What software, if applicable, was used to manage the data? | NVivo 9 was used for data management and analysis. |
|  | Participant checking | Did participants provide feedback on the ﬁndings? | No |
|  | *Reporting* |  |  |
|  | Quotations presented | Were participant quotations presented to illustrate the themes / ﬁndings? Was each quotation identiﬁed? e.g. participant number | Yes |
|  | Data and ﬁndings consistent | Was there consistency between the data presented and the ﬁndings? | Yes, we have attempted to present our findings in this work clearly in a manner that was consistent with the data collected. |
|  | Clarity of major themes | Were major themes clearly presented in the ﬁndings? | Yes, the findings are presented in the result section. |
|  | Clarity of minor themes | Is there a description of diverse cases or discussion of minor themes? | NA |
