# Supplementary material for: SMART Mental Health Project: process evaluation to understand the barriers and facilitators for implementation of multifaceted intervention in rural India
Source: Int J Ment Health Syst. 2021 Feb 8;15:15. doi: 10.1186/s13033-021-00438-2 (PMC7871593; doi:10.1186/s13033-021-00438-2)
Supplement: Supplementary file 3 — Additional file 3. Key themes and reflective quotes. [file 13033_2021_438_MOESM3_ESM.docx]

**Additional Table 3: Key themes and Reflective quotes**

| S.No. | **Facilitator** | **Reflective Quotes** |
| --- | --- | --- |
|  | Appraisal of the programme and related activities | - *“The program was very good…90% useful. One drama was shown to us as a part of the program. It was really good, and people understood how a person suffers from mental disorders. We all felt very happy and people changed a lot”. (Village leader, IDI- 8)* - *“Your people [field staff] came to each and every house, asked questions, collected information, and explained about the program. They [field staff] asked everyone about their health and related issues”. (Key village member, IDI-23)* - *“The way people [project staff] approached was very good. If somebody persuades about something definitely anyone will respond positively. Because your [project staff] people are continuously coming and enquiring, villagers are expressing themselves and some of them are getting relieved from their problems. They [project staff] are very good” (Community member, FGD-5)* - *“We felt that at least someone [ASHAs] came and enquired about our well-being. We got someone [ASHAs] to share our problems which may reduce our pain.” (Screen positive –visited doctors for treatment, FGD-4)* - *“We always welcome your health programmes. These programmes should be continued forever. People shall always be in need of a psychiatrist because some (or) other shall always face some problem regularly. They get depressed always with difficulties and they need someone to lift them up”. (Community leader, IDI-9)* - *“Yes, the programme was useful. For regular health problems, people go to hospital but they never try to go to hospital for these [mental health] problems. So, if programs are conducted on mental health issues, people will get an understanding about mental health problems, and may go to a doctor”.(Village Head, IDI-8)* |
|  | Benefits of trainings | - *“Training was very good, we learnt how to follow up the patients, in what way we have to respond to people, elders or others in the village. We are giving the information what they are requesting. We learned many new things during training. So now we don't have any issues in field”. (Field staff, FGD-8)* - *“It was easy for us to deal with patients as everything was put on the tab, time was saved and work was fast otherwise have to wrote everything on the paper”.( Doctor, IDI-3)* |
|  | Benefits of using EDSS | - *“This program helped us professionally to improve our counselling skills and how to spend more time with patient and how to speak with patients. In this way we are improving our skills as a doctor”. (Doctor, IDI-4)* |
|  | Anti-Stigma Campaign and Increase in Awareness of CMDs and its treatment | - *“Interacting with the patient and getting information about their health is really good. Actually these people don't know that they have CMD they imagine it differently …as mad …as abnormal”. (Doctor, IDI 03)* - *“Common people can’t identify mental issues. We only think insane people are mental patients. I too had such feeling but when I took one month training, I learned about the mental health issues. When I went to field for the first time, most of them said they don't have any mental problem. Later when we went to Baseline and post intervention, then they started recognizing those issues”. (Field staff, FGD-8)* |
|  | Preference for awareness campaign strategy | - *“Door to door campaigning is better because everyone get information and knowledge about mental health.” (Community members, IDI-13)* |
|  | Engagement of ASHA workers | - *“Yes, definitely they [community members] pay attention to ASHA. Ladies mostly approach ASHA only whatever be their problem. ASHA only takes care of ladies all through the pregnancy. Once the child is delivered they take care of vaccines and other things. So, definitely everyone listen to ASHA with a trust. (Community Member, FGD-1)* - *“People won’t speak openly with an outsider. ASHA is a native person and they feel comfortable to share everything with her [ASHA]” (Community member, FGD-3)* - *“ASHA workers are really good. They persuade us to go to doctor and sometimes they accompanied us also to visit the hospital.”(Community member, FGD-4)* |
|  | Easy access of treatment in vicinity | - *“Definitely these [medical] camps were useful. If doctors come voluntarily, check everyone and provide treatment for the patients…it is really good”. (Community member, FGD-10)* - *“Actually, in my general OPD, I get 90-100 patients. But this [camp] is totally different. I saw so many patients who are not willing to come to PHC are coming here. Dealing with CMD patients is totally different from general patients”. (Doctor, IDI-5)* - *“When we used to hear their problems we felt that our problems are very less and we tried to handle our problems and helped them also to come out of them”(ASHA FGD)* |
|  | **Barriers** | **Reflective Quotes** |
|  | *Limited awareness on mental illness* | - *“They [community members] might be thinking that mental health problems means like a contagious disease and suffering from that on which the day starts”. (Community member, FGD-4)* - *“Sometimes we tell our problems to friends and family members but normally people would not consider their problem as a serious one.” (Community member,FGD-11)* |
|  | *Stigma associated with mental illness* | - *“In my family also people won’t agree to go to a hospital. Now, I never told them that I am coming here for treatment. They already shouted at me for talking with Asha workers and for taking doctor appointment” (Screen positive-visited the doctor,IDI-15)* - *“Most of the people did not come as it [mental illness]is a social stigma and [other]people may consider them as mad though our staff educated them and tried to bring them but a few never turned up. They wanted to meet the doctor secretly and get some medicine” (Doctor,IDI-2* ) - *“They [community member] fear that other people[will] think they have mental [health]issues and they are mad. Many people have these perceptions”. (ASHAs, FGD-2)* |
|  | *Accessing health care: Distance, financial and Socio-Cultural constraints* | - *“With transportation issues, some people are not going. For example, one village is there. We need to walk 3kms to come out of the village, from where they have to go to another village and walk another 1km to reach PHC, so because of these transportation issues some people are not going.”(ASHA, FGD-5)* - *“We were surprised that, these people are saying these things in detail. We also felt that, it is good if you give medicines along with that”(Screen Positive_visited_doctor,FGD-2)* - *“So many females in village are going to Dubai and earning so much. They [men] are getting addicted all sorts of bad habits. This [alcoholism] is also one of the main reasons for the male members to develop CMD.”(Village head,IDI-9)* - *“Women won't get any work.They are suffering by simply sitting at home. Their husbands spend all their earned money on drinking[alcohol] and give less money to the family. With this situation, they go into depression and think of committing suicide”. (ASHA, FGD -2)* |
|  | *Usefulness of Voice/text messages* |  |
|  | *EDSS/IVRS Application* | - *“Signals…very poor at time. Sometimes they[data] are not synchronising. So many times we faced inconvenience because of this synchronizing process. The data done by ASHA’s is not synchronize into our tabs. It took so many days for that to be sorted out”. (Doctor, IDI-4)* - *“Villagers who were not familiar with number will think it is company message and ignore it” .(ASHA-FGD-2)* - *No, I don’t have any idea about messages. I never checked whether I got or not.*   *I saw a few messages. I don’t know what messages they are I never paid much interest”.(Community Members, FGD-3)*   - “ *I don’t think people will listen. Because so many are ignoring messages from prime minister/chief minister, ordinary messages mean at least educated people will read. Voice messages I think won’t be useful”.(Community Member, FGD 2)* - *“No normally I’ll give my husband number only. He must be getting messages. I don’t know”.(Community Members, FGD1)* |
|  | **Recommended improvements to programme** | **Reflective Quotes** |
|  | *Need to create awareness on mental health issues* | - *“It is very important to conduct review meetings…every fort night or month. So that we can get an exact idea about the status of the patients and make our services better.”(Doctor, IDI- 5)* - *“Now a days after television] entered our life, everybody knows everything. Sometimes people take decision on the basis of information received through this[television] channel, similarly such[mental health] kind of activities need to be advertised much more.”(Screen positive not visited doctor, FGD-2))* |
|  | *Social Support* | - *“Nobody will come [for medical camp] as they are not particular about mental health. If camps are conducted for heart problems, ENT problems or for any other health problem, definitely they will come. For mental disorders, they won’t come because they don’t know at all that they have a problem.”(Village leader,IDI-11)* - *“Now there are 104. For BP [blood pressure], sugar [diabetes] almost every month they use medicines. There are nearly 80 members. Daily people will come and use the medicines. Like that they will increase here too. Like that, if there is any benefit for them from PHC, and they get medicines then no one will stop coming”.* - *“Husband and children should know and understand that their wife/ mother’s mental health condition and should be supportive. (Screen positive visited doctor, FGD- 5)* |
|  | *Additional Trainings are required* | - *“the training should be provided for at least entire one day at regular intervals, and follow up classes should be conducted at least once in a month.” (Doctor,IDI-1)* - *“George Institute can support in a PPP [public private partnership programme] which is a public private partnership by explaining the SMART Health programme and its objective.” (Government Official IDI-1)* |
